# Social Memory and Social Patterns Alterations in the Absence of STriatal-Enriched Protein Tyrosine Phosphatase

**DOI:** 10.3389/fnbeh.2018.00317

**Published:** 2019-01-25

**Authors:** Gloria Blázquez, Anna Castañé, Ana Saavedra, Mercè Masana, Jordi Alberch, Esther Pérez-Navarro

**Affiliations:** ^1^Departament de Biomedicina, Facultat de Medicina i Ciències de la Salut, Institut de Neurociències, Universitat de Barcelona, Barcelona, Spain; ^2^Institut d’Investigacions Biomèdiques August Pi i Sunyer (IDIBAPS), Barcelona, Spain; ^3^Centro de Investigación Biomédica en Red sobre Enfermedades Neurodegenerativas (CIBERNED), Barcelona, Spain; ^4^Department of Neurochemistry and Neuropharmacology, CSIC-Institut d’Investigacions Biomèdiques de Barcelona (IIBB), Barcelona, Spain; ^5^Centro de Investigación Biomédica en Red de Salud Mental (CIBERSAM), Barcelona, Spain

**Keywords:** social memory, social interaction, dominance, STEP KO mice, dopamine

## Abstract

STriatal-Enriched protein tyrosine Phosphatase (STEP) is a neural-specific protein that opposes the development of synaptic strengthening and whose levels are altered in several neurodegenerative and psychiatric disorders. Since STEP is expressed in brain regions implicated in social behavior, namely the striatum, the CA2 region of the hippocampus, cortex and amygdala, here we investigated whether social memory and social patterns were altered in STEP knockout (KO) mice. Our data robustly demonstrated that STEP KO mice presented specific social memory impairment as indicated by the three-chamber sociability test, the social discrimination test, the 11-trial habituation/dishabituation social recognition test, and the novel object recognition test (NORT). This affectation was not related to deficiencies in the detection of social olfactory cues, altered sociability or anxiety levels. However, STEP KO mice showed lower exploratory activity, reduced interaction time with an intruder, less dominant behavior and higher immobility time in the tail suspension test than controls, suggesting alterations in motivation. Moreover, the extracellular levels of dopamine (DA), but not serotonin (5-HT), were increased in the dorsal striatum of STEP KO mice. Overall, our results indicate that STEP deficiency disrupts social memory and other social behaviors as well as DA homeostasis in the dorsal striatum.

## Introduction

STriatal-Enriched protein tyrosine Phosphatase (STEP) is a neural-specific phosphatase that opposes the development of synaptic strengthening through the regulation of multiple kinases and glutamate receptor subunits critical for synaptic plasticity. It acts by dephosphorylating the GluN2B and GluA2 regulatory subunits of NMDA and AMPA receptors, respectively, leading to their internalization, and it promotes synaptic weakening by dephosphorylating the regulatory tyrosine (Tyr) of ERK1/2, Fyn or Pyk2 kinases resulting in their inactivation (Goebel-Goody et al., 2012a). Its dysregulation has been reported in several psychiatric and neurodegenerative diseases (Goebel-Goody et al., 2012a; Karasawa and Lombroso, 2014), and preclinical reports indicate that genetic deletion or pharmacological inhibition of STEP improves cognitive deficits in mouse models of Alzheimer’s disease (Zhang et al., 2010; Xu et al., 2014), fragile X syndrome (Goebel-Goody et al., 2012b; Chatterjee et al., 2018) and schizophrenia (Xu et al., 2018), as well as age-related memory decline (Castonguay et al., 2018).

STEP is highly expressed in the striatum and at lower levels in the cortex, hippocampus, amygdala and other brain regions, except in the cerebellum (Lombroso et al., 1991, 1993; Boulanger et al., 1995). The striatum plays a role in the computation of social behavior (van den Bos, 2015), being implicated in those behaviors that occur in a social context, like social reward behaviors and learning in social contexts (Báez-Mendoza and Schultz, 2013). The amygdala works as a hub to modulate a variety of brain networks that are important to normal social cognition (Bickart et al., 2014), and the cortex participates in the social reasoning (Bault et al., 2011). In the hippocampus, STEP is enriched in the CA2 (Shinohara et al., 2012; Kohara et al., 2014), a crucial region for socio-cognitive memory processing (Hitti and Siegelbaum, 2014; Stevenson and Caldwell, 2014), as well as for the modulation of further social patterns such as aggressive behavior (Pagani et al., 2015). It was previously reported that, like wild-type (WT) mice, STEP knockouts (KOs) show preference for a novel than for a known mouse, which was interpreted as mutant mice having intact social memory (Venkitaramani et al., 2011). Contradictorily, in another work, neither WT nor STEP KO mice spent more time exploring the novel mouse compared to the familiar one in the three-chamber sociability test (Goebel-Goody et al., 2012b) pointing at some procedural artifact. Given these controversial results and STEP expression profile, in the present work we sought to examine the role of STEP in social memory and further social patterns by thoroughly characterizing the social phenotype of STEP KO mice.

## Materials and Methods

### Animals

Six months old male (C57BL/6J background) STEP KO (Venkitaramani et al., 2009) and WT mice were housed in groups of 2–5 animals per cage, maintained under standard housing conditions, 12 h light/dark schedule (lights on at 08:00 am) with food and water *ad libitum*, 22 ± 2°C room temperature and 50%–70% humidity. Mice were habituated to handling and given 1 h to habituate after transport to room before any tests were conducted. Experimental procedures were approved by the Local Ethical Committee of the University of Barcelona following European (2010/63/UE) and Spanish (RD53/2013) regulations for the care and use of laboratory animals. After behavioral assessment mouse genotype was confirmed by Western blot analysis (Supplementary Figure S1).

### Behavioral Tests

Social memory and further social patterns were assessed by using the behavioral battery described below. Moreover, non-social memory abilities, sensorimotor and olfactory capabilities as well as anxiety levels were also studied to analyze their possible influence on social patterns. Two batches of animals were used, and the experimental timeline of the tests, from less to more aversive (McIlwain et al., 2001), is depicted in Supplementary Figure S2. Animal behavior was videotaped and analyzed using the SMART v3.0 software (Panlab, Barcelona, Spain). When appropriate, the arenas were cleaned with 5% ethanol between trials to remove any odor cues (Blázquez et al., 2014).

### Three-Chamber Sociability Test

Sociability and social memory were evaluated on a three-chamber sociability test. Subjects were first habituated to the empty apparatus, a three-chamber box consisting of three interconnected lined compartments (DeVito et al., 2009) with open doors, for 10 min trial/day, for 3 days. Age and sex-matched mice to be explored were also habituated for 3 days to be caged in jails inside the apparatus. On the testing day, subject mice were habituated to the central compartment with closed doors for 5 min. After the habituation phase, subjects were tested in the sociability task, and 20 min later the social memory task was performed to evaluate preference for social novelty. The sociability task consisted in giving the subject mice the option to socialize with a conspecific mouse or explore a mouse dummy located in opposite external compartments. The social memory task performed 20 min later consisted in presenting to subjects, in the opposite compartment respect to the initial encounter, the known mouse (same as during the sociability phase) and a stranger mouse in the other external compartment. The trial tests lasted for 10 min and distance traveled, number of entrances, time spent in each compartment and time sniffing each cage were measured (DeVito et al., 2009).

### Social Discrimination Test

Social memory was evaluated in the social discrimination test (Engelmann et al., 2011). Subjects were habituated to be individually caged in standard clean cages for 2 h before being tested. A mouse juvenile (C57BL/6J, 15–35 days old) was introduced in the subject’s cage during a 4 min trial for the sampling phase. After a 1 h inter-trial interval (ITI) a 4 min choice phase was performed in which the previously encountered juvenile was presented to the subject. After another 1 h ITI, a new choice trial was performed in which a novel juvenile was introduced into the subject’s cage. Interaction time (including anogenital and nose-to-nose sniffing as well as allogrooming) was evaluated, and the difference between time spent in social interaction during the sample and the choice phases was scored.

### 11-Trial Habituation/Dishabituation Social Recognition Test

Social memory was also assessed in the 11-trial habituation/dishabituation social recognition test. Subject male mice were habituated to a clean standard home cage for 5 h before the test. Age-matched C57BL/6J females in metestrus and diestrus estrous cycle were selected as stimulus for the social memory test (see procedure below). Female A and B belonged to different cages. The test consisted in a habituation phase where female A was presented to subject male for 10 trials of 1 min each, and a 10 min ITI. In the 11th trial, an unknown female B was presented to subject male in the test phase. Interaction time (including anogenital and nose-to-nose sniffing, as well as allogrooming) was evaluated and the difference between time spent in social interaction during the habituation phase and the test phase was scored (Fergusson et al., 2000; Stevenson and Caldwell, 2014). Females (*n* = 8) were vaginal washed with 25 μl PBS flushed 4–5 times with a pipette tip introduced 1 mm in the vagina, until getting a turbid solution. One drop of the smear was put on a microscope slide, and once air dried it was stained by submersion during 3 min in a 0.1% crystal violet solution (Scharlau S.L., Spain), and rinsed twice during 1 min in distilled water. This protocol was repeated daily during a week, and only females in metestrus and diestrus were used as subject of interest for tested males (Supplementary Figure S3; McLean et al., 2012). Each female was presented just once a day to a subject male.

### Novel Object Recognition Test (NORT)

Hippocampal-dependent learning and memory was analyzed using the novel object recognition test (NORT; Dere et al., 2007). Mice were first habituated to the arena (square white box: 59 cm lateral × 40 cm height) in the absence of objects, for 2 trials of 10 min duration with an ITI of 4 h. The second day, a training session was performed during 10 min by presenting two similar objects resembling eggs. After a 15 min ITI the testing session was performed, in which subjects were exposed for 5 min to a familiar (egg-like) and a new object (a plug). The object preference was measured as the time exploring each object × 100/total time exploring.

### Olfactory Habituation/Dishabituation Test

Olfactory capabilities were assessed in the olfactory habituation/dishabituation test to elucidate if mice were able to smell and distinguish different social odors (Yang and Crawley, 2009). Subjects were first habituated to be individually housed in clean home cages 1 h before the experiment took place, and a wire ball (tea container of 2 cm diameter) containing a piece of cotton was introduced for a 30 min habituation. The test consisted in sequential presentations of non-social odor (clean bedding) and two different social odors that were obtained by impregnating a piece of cotton with dirty bedding (7 days old, from home cage of five male mice). Each odor was presented for three consecutive 2 min duration trials. The ITI was 1 min, the time needed to change the odor stimulus. Habituation was defined as the progressive decrease in olfactory investigation towards a repeated presentation of the same odor stimulus. Dishabituation was defined by a reinstatement of sniffing when a novel odor was presented. Time sniffing the wire ball was scored.

### Sensorimotor Battery

The sensorimotor capabilities were evaluated by a SHIRPA standard task battery (Rogers et al., 1997) including motor coordination and equilibrium assessed in the iron rod and in the wire hanger tests, and prehensility and muscular strength in the wire hanger test, as previously described (Blázquez et al., 2014).

### Nesting Behavior and Group Sleeping

Nesting behavior was assessed by using a protocol modified from Deacon (2006). Group-housed mice were transferred to new home cages with two pieces of soft paper for nesting, and nests were assessed 24 h later on a rating scale of 1–5. Group sleeping was scored 1 and individual sleeping was scored 0. Data was analyzed by a Fisher’s test.

### Corner Test

Neophobia or fearfulness to novelty was assessed in the corner test. Subjects were individually introduced in the center of a clean home cage (sides of 23 cm), and exploratory behavior was assessed as the number of rearings and corners explored during 30 s (Blázquez et al., 2014).

### Open Field

To assess exploratory activity influenced by fearfulness to a novel environment, mice were individually placed in the center of an open round arena located in the center of an illuminated room. The open field apparatus was a white wooden arena of 38.5 cm diameter. Latency to initiate movement (initial freezing), distance traveled in cm, number of rearings and defecation boluses were measured in a single 5 min trial (Blázquez et al., 2014).

### Plus Maze

Anxiety levels were assessed in the plus maze. The apparatus consisted of two opposing open arms (58 × 8 cm) crossed by two opposing enclosed arms (58 × 8 × 12 cm), and an open 8 × 8 cm square in the center. The maze was made of black plexiglass, and was elevated 50 cm above the floor. Mice were placed in the center of the plus maze facing an enclosed arm and behavior was measured during 5 min. The latency, number of entries, distance traveled into the open and closed arms, and number of defecation boluses were measured (Fernández-Teruel et al., 2002).

### Dark-Light Box

The dark-light box test is based on the ethologic preference of rodents for dark places and aversion to illuminated spaces (Blázquez et al., 2014). The apparatus consisted of two compartments {black/dark: 13 × 14 × 27 cm; white/illuminated [with a white light bulb (390 luxes)]: 16 × 14 × 27 cm} separated by a wall with an opening (7 × 7 cm) that connected both spaces. Latency to the first entry into the white compartment, total number of entries and distance traveled into the white compartment were measured in a 5 min session.

### Tail Suspension Test

The tail suspension test was used to evaluate behavioral despair (Can et al., 2012; Ye et al., 2016) by measuring time in immobile posture when mice were subjected to the short inescapable stress of being suspended by their tail. The mouse tail was introduced in a plastic cylinder (4 cm length × 1.3 cm diameter) to avoid tail climbing, and tape was subjecting 1–2 cm of the tail tip, suspending the mouse from the top of a white square plexiglass box (60 cm sides × 40 cm depth, one side open to see the mouse), 60 cm above the floor. Latency to immobility and immobility time (none of the four paws moving) were measured during a 6 min trial.

### Dominance Tube Test

Subjects were tested in a tube test for social dominance assessment (Lijam et al., 1997; Spencer et al., 2005). A transparent plexiglass 35 cm length × 3.5 cm diameter tube was used. After training the animals to cross the tube the day before, two subjects of different genotype were released simultaneously on opposite sides of the tube for a maximum of 2 min encounter. The match ended when one of the mice completely retreated from the tube. The subject remaining in the tube was the winner, scoring 1 point, and the retreated subject was scored with 0 points. Each mouse was matched in three trials with three different subjects of the opposite genotype.

### Resident-Intruder Test

Social interaction was evaluated in the resident-intruder test as previously described (Lumley et al., 2000; Wood and Morton, 2015), with some modifications. The test had three steps: habituation, barrier and interaction phases. For the habituation phase, all mice but the “resident” were removed from their home cage (23 cm sides, containing dirty bedding from a few days to establish the territory). A wire net was introduced in the middle of the home cage dividing it into two equal spaces. The subject mouse was left for 5 min in one of the spaces to adapt to the barrier. Time interacting with the barrier was measured. After the habituation phase, an unknown C57BL/6J age-matched male intruder mouse was introduced into the other space for 5 min. Time interacting with the intruder in the presence of the barrier was scored in this phase. Finally, the barrier was removed and resident mouse could interact directly with the intruder. Time of interaction was measured during a 5 min trial.

### *In vivo* Microdialysis

Extracellular serotonin (5-HT) and dopamine (DA) levels were measured by *in vivo* microdialysis as previously described (Castañé et al., 2008). Briefly, one concentric dialysis probe equipped with a Cuprophan membrane (1.5 mm long) was implanted in the dorsal striatum of anesthetized mice (sodium pentobarbital, 40 mg/kg, i.p.) at coordinates (in mm, from bregma and skull): AP +0.5; L −1.7; DV −4.5 (Franklin and Paxinos, 1997). Microdialysis experiments were performed in freely moving mice 24 h (day 1) and 48 h (day 2) after surgery. The artificial cerebrospinal fluid (aCSF) was pumped at 1.65 μl/min, and dialysate samples were collected every 20 min in microvials containing 5 μl of 10 mM perchloric acid. Following an initial 30 min stabilization period, six baseline samples were collected before local (reverse dialysis) veratridine (50 μM) or nomifensine (1, 10 and 50 μM) administration on day 1 and day 2, respectively. Veratridine (Tocris; Bristol, UK) was dissolved in dimethyl sulfoxide 99.9% (Sigma-Aldrich, Tres Cantos, Spain) to 5 mM (stock solution). Nomifensine maleate salt (Sigma-Aldrich) was dissolved in aCSF to 1 mM (stock solution). Stock solutions were stored at −20°C until use, and working solutions of veratridine and nomifensine were prepared by dilution in aCSF. 5-HT and DA concentration was analyzed by HPLC with amperometric detection at +0.6 V and +0.7 V, respectively, with a detection limit of 2 fmol/sample. Following sample collection, mice were sacrificed and brains were removed, sectioned and stained with neutral red to ensure proper probe placement.

### Western Blot Analysis

Western blot analysis was performed as previously described (Saavedra et al., 2011). The primary antibodies used were: anti-STEP (1:1,000; Santa Cruz Biotechnology, Santa Cruz, CA, USA), anti-DA D1 receptor (D1R; 1:500; Cell Signaling, Bevelly, MA, USA) and anti-DA D2 receptor (D2R; 1:1,000, Frontier Institute, Japan). Loading control was performed by reprobing the membranes with an anti-α-tubulin antibody (1:50,000; Sigma-Aldrich) for 15–20 min at room temperature. Then, membranes were washed with Tris-buffered saline containing 0.1% Tween 20 (TBS-T), incubated for 1 h (15–20 min for loading controls) at room temperature with the corresponding horseradish peroxidase-conjugated secondary antibody (1:2,000; Promega, Madison, WI, USA), and washed again with TBS-T. Immunoreactive bands were visualized using the Western Blotting Luminol Reagent (Santa Cruz Biotechnology, Santa Cruz, CA, USA), and quantified by a computer-assisted densitometer (Gel-Pro Analyzer, version 4, Media Cybernetics; Warrendale, PA, USA).

### Statistical Analysis

Data are presented as mean ± SEM. Behavioral data were analyzed using the software SPSS Statistics 22, and biochemical data using the GraphPad Prism (v. 5.01, GraphPad Software Inc., San Diego, CA, USA). The statistical test applied in each experiment is detailed in the text/figure legends. Statistical significance was set at 95% confidence level.

## Results

### STEP KO Mice Display Impaired Social Memory

**Figure 1:**
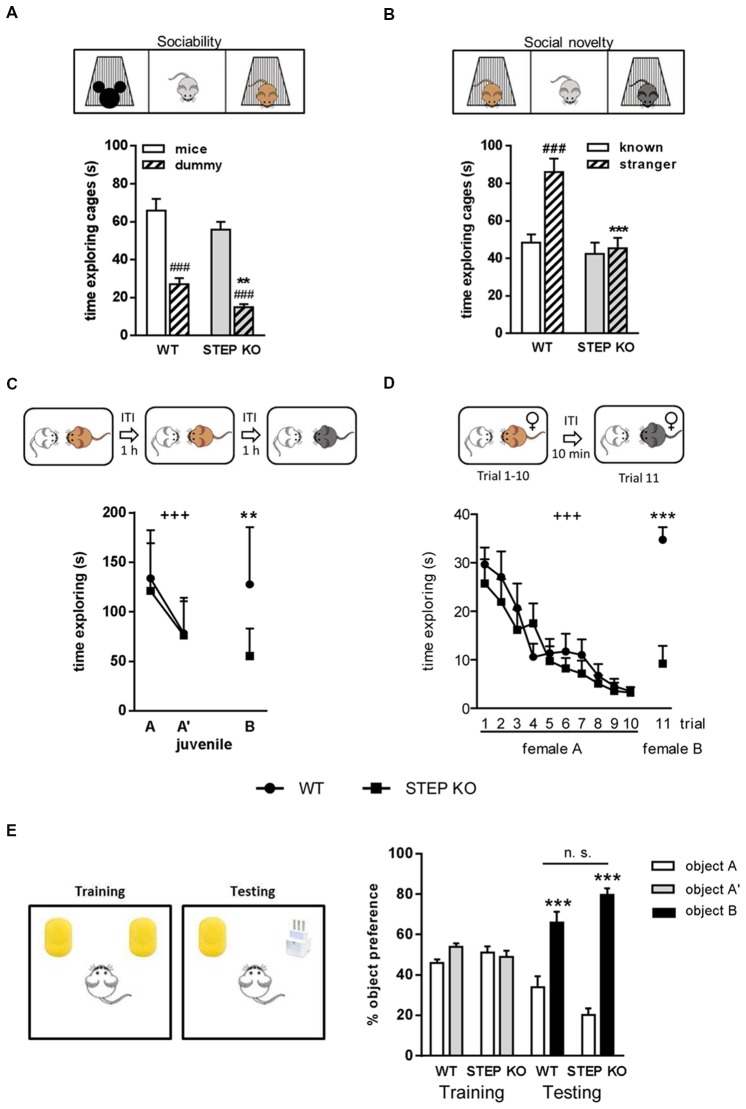
Lack of STriatal-Enriched protein tyrosine Phosphatase (STEP) produces impairments in social memory. **(A)** Time exploring the mouse and dummy cages during the socialization in the three-chamber sociability test in wild-type (WT) and STEP knockout (KO) mice. Two-way ANOVA was performed indicating “genotype” and “cage” effect. ^###^*p* < 0.001, “cage” effect for each genotype (Student’s dependent *t*-test), **p < 0.005 “genotype” effect when analyzing the time exploring the dummy cage. **(B)** Time exploring the known and the stranger mouse cages in the short-term memory evaluation of the three-chamber sociability test in WT and STEP KO mice. Two-way ANOVA was performed indicating “genotype” and “cage” effect. ^###^*p* < 0.001, “cage” effect (Student’s dependent *t*-test), ***p < 0.001 “genotype” effect when analyzing the time exploring the stranger mouse cage. **(C)** Time exploring the same juvenile in the first two trials, and a new juvenile in the third trial of the direct interaction test in WT and STEP KO mice. ^+++^*p* < 0.001, “trial” effect [repeated measures ANOVA (MANOVA)], **p < 0.005 “genotype” effect (Student’s independent *t*-test). **(D)** Time exploring the female A during the first 10 trials, and a new female B in the 11th trial of the 11-trial social memory test in WT and STEP KO mice. ^+++^*p* < 0.001, “trial” effect (repeated MANOVA), ***p < 0.001 “genotype” effect (Student’s independent *t*-test). Results are presented as mean ± SEM [*n* = 11–12 for **(A,B)**; *n* = 9–10 for **(C,D)**]. **(E)** Percentage of time exploring each object during the training and testing phases of the novel object recognition test (NORT). Results are represented as mean ± SEM (*n* = 10–12). “Genotype” effect n.s., ***p < 0.001 “object” effect (two-way ANOVA).

To evaluate the effect of genetic deletion of STEP on social memory we first subjected WT and STEP KO mice to the three-chamber sociability test. During the socialization phase, STEP KO mice traveled less distance than WT mice (Student’s *t*-test, WT: 3486.23 ± 135.69 cm and STEP KO: 3015.04 ± 111.69 cm, *t*_(1,21)_= 2.653, *p* < 0.05), showing diminished levels of exploration compared to controls. Moreover, although WT and STEP KO mice spent more time exploring the mouse cage than the dummy cage (intragenotype comparison, Student’s *t*-test, WT: *t*_(1,16.64)_= 5.79, *p* < 0.001; STEP KO: *t*_(1,12.84)_ = 9.33, *p* < 0.001), indicating comparable levels of sociability and similar time spent for memory acquisition (Figure 1A), total exploration time of both cages was lower in STEP KO mice compared to WT group (WT: 92.99 ± 7.64 s and STEP KO: 70.71 ± 3.48 s, Student’s *t*-test, *t*_(1,15.31)_ = 2.65, *p* < 0.05). When we analyzed social memory 20 min later, STEP KO mice showed reduced traveled distance (WT: 3831.05 ± 95.78 cm, STEP KO: 2895.38 ± 143.81 cm, Student’s *t*-test, *t*_(1,21)_ = 5.50, *p* < 0.001), and less exploration of both cages (WT: 134.50 ± 8.62 s, STEP KO: 87.54 ± 8.24 s, *t*_(1,21)_ = 3.92, *p* < 0.001) compared to WT group. Importantly, while WT mice displayed social memory and spent more time exploring the “stranger” mouse than the “known” mouse cage (Student’s *t*-test, *t*_(1,22)_ = 4.46, *p* < 0.001), STEP KO mice showed no preference for any mouse cage (Student’s *t*-test, *t*_(1,20)_ = 0.37, n.s.) pointing at social memory alterations (Figure 1B).

To further characterize social memory performance of STEP KO mice, we next used the social discrimination test. When the same juvenile was presented in the second trial (A’), mice from both genotypes showed a habituation effect (repeated measures ANOVA (MANOVA) “trial”: *F*_(1,17)_= 39.42, *p* < 0.001), without differences between genotypes (repeated MANOVA “genotype × trial,” *F*_(1,17)_ = 0.67, *p* = 0.42; “genotype,” *F*_(1,17)_ = 0.34, *p* = 0.56; Figure 1C). In the third trial, when a new juvenile (B) was presented to the subject mouse, STEP KO mice explored the unknown mouse at similar levels as the previous habituation trial, whereas control mice explored longer, as during the first trial (Student’s *t*-test “genotype” effect, *F*_(1,18)_= 11.93, *p* < 0.005). Comparison between the first and the third trial also showed differences between genotypes (ANOVA, WT: 9.95 ± 17.33 s, STEP KO: 65.99 ± 11.73 s, *F*_(1,18)_ = 6.85, *p* < 0.05) in the dishabituation, thus indicating social memory alterations.

We also performed the 11-trial habituation/dishabituation social recognition test. Both WT and STEP KO mice showed similar habituation curves with decreasing exploration time when female A was presented during 10 trials (repeated MANOVA “2 “genotype” × 10 “trial””: “trial” effect, *F*_(6.38,108.55)_= 18.16, *p* < 0.001; “genotype” effect, *F*_(1,17)_ = 0.391, *p* = 0.54). However, when a new female was presented to the subject mouse in the 11th trial there were differences between genotypes (*F*_(1,18)_= 34.45, *p* < 0.001) since WT animals spent significantly more time exploring the new female than in the previous trials with the known one, which was not the case for STEP KO mice (Figure 1D). Altogether, these results showed that lack of STEP activity impairs social recognition memory.

To determine whether STEP KO have a general recognition memory deficit we evaluated their object recognition memory using the NORT. In the first habituation the traveled distance was similar in WT and STEP KO mice (Student’s *t*-test, WT: 4178.57 ± 320.38 cm and STEP KO: 3493.39 ± 215.42 cm, *t*_(1,21)_ = 1.74, n.s.), while in the second habituation STEP KO mice traveled less distance than WT mice (Student’s *t*-test, WT: 2534.48 ± 119.92 cm and STEP KO: 1638.02 ± 155.24 cm, *t*_(1,21)_ = 4.61, *p* < 0.001). No differences in object preference were found during the training phase when two identical objects were presented (ANOVA, “genotype” effect, *F*_(1,21)_ = 1.11, n.s.; “object” effect, *F*_(1,21)_ = 0.72, n.s.) although STEP KO mice explored less time both objects than WT mice (Student’s *t-test* WT: 21.38 ± 3.32 s and STEP KO: 11.91 ± 2.78 s, *t*_(1,21)_ = 2.16, *p* < 0.05). Both genotypes explored longer the new object than the known one in the testing phase (ANOVA, “genotype” effect,*F*_(1,20)_ = 2.58, n.s.; “object” effect, *F*_(1,20)_= 48.76, *p* < 0.001) and there were no significant differences in time of exploration of both objects (Student’s *t*-test WT: 30.35 ± 5.74 s and STEP KO: 16.72 ± 4.99 s, *t*_(1,21)_ = 1.17, n.s.), indicating similar levels of object recognition memory (Figure 1E).

### STEP KO Mice Have Unaltered Olfactory Function

**Figure 2:**
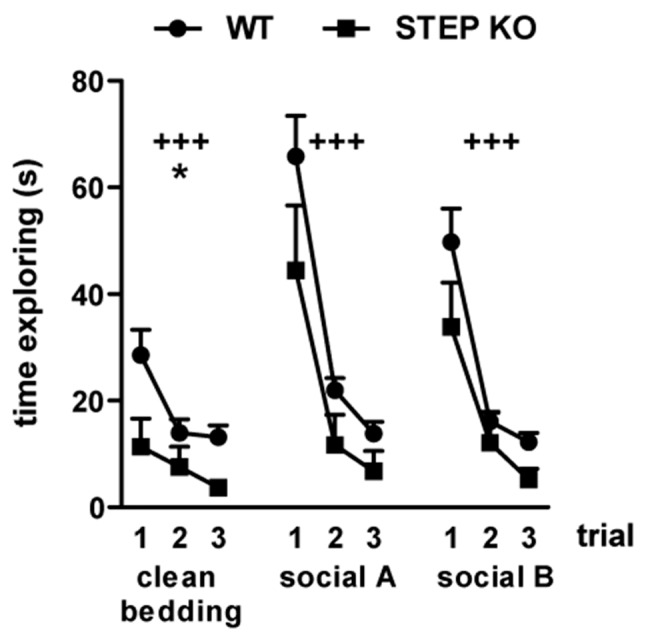
STEP KO mice have normal olfactory function. Time exploring three different scents including clean bedding and two different social odors (social A and social B) in the olfactory habituation/dishabituation test. Results from WT and STEP KO mice are presented as mean ± SEM (*n* = 9–10). ^+++^*p* < 0.001, “trial” effect, *p < 0.05 “genotype” effect (repeated MANOVA).

Given that rodent social interactions largely depend on a functional olfactory system (Ropartz, 1968; Matochik, 1988; Popik et al., 1991), we asked whether STEP KO mice have affectations in the olfactory function. To address this possibility, WT and STEP KO mice were subjected to the olfactory habituation/dishabituation test. Mice from both genotypes showed habituation when each scent was presented during three consecutive trials (repeated MANOVA 3 “trial” × 2 “genotype,” clean bedding: “trial” effect *F*_(1,34)_= 10.61, *P* < 0.001, “genotype” effect *F*_(1,17)_ = 7.39, *p* < 0.05; social odor A: “trial” effect *F*_(1.38,23.50)_= 50.65, *p* < 0.001; social odor B: “trial” effect *F*_(1.71,29.06)_= 39.01, *p* < 0.001; Figure 2). There was also dishabituation when a new scent was presented, without differences between genotypes (Student’s *t*-test “trial 1 social odor A—trial 3 clean bedding,” WT vs. STEP KO mice: *t*_(1,17)_ = 1.45, n.s.; “trial 1 social odor B—trial 3 social odor A,” WT vs. STEP KO mice: *t*_(1,17)_ = 0.84, n.s.; Figure 2), thus indicating that STEP KO mice have intact smell sense, and are able to distinguish different social odors.

### Lack of STEP Has No Effect on Sensorimotor Functions or Anxiety Levels

**Figure 3:**
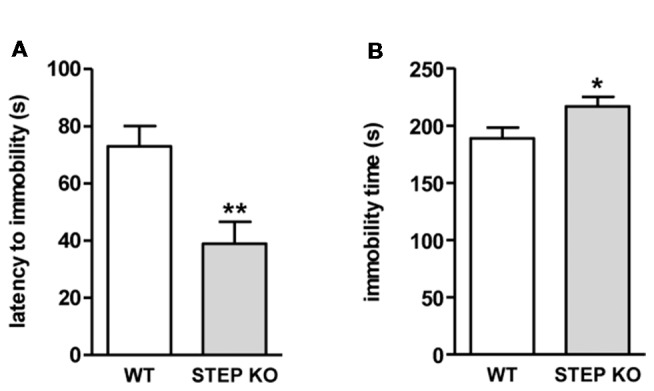
STEP KO mice present behavioral despair. **(A)** Latency to first movement and **(B)** accumulated time of immobility in WT and STEP KO mice in the tail suspension test. Results are represented as mean ± SEM (*n* = 11–12). *p < 0.05, **p < 0.01 (Student’s independent *t*-test).

To know if the alterations found in social memory in STEP KO mice may be in part influenced by sensorimotor alterations and/or changes in anxiety levels, the animals were tested to assess these phenotypes. STEP KO mice did not show alterations in visual acuity, auditory reflex or escape reflex when assessed in a SHIRPA battery (data not shown). Nesting behavior, group sleeping, physical appearance, and absence of hind limb clasping were similar in both genotypes. As represented in Table 1, STEP KO mice did not show motor alterations in the wire hanger test or in the iron rod test, but they explored fewer corners than WT mice in the corner test. In line with the diminished exploratory levels seen in the corner test, STEP KO mice traveled less distance than WT mice in the open field (Supplementary Figure S4), performing more grooming behavior, and presenting more defecation boluses (Table 1). To assess possible alterations in anxiety levels, mice were tested in the plus maze and dark-light box. Data showed that both WT and STEP KO mice presented similar levels of activity in the more aversive areas, the open arms of the plus maze and the light compartment of the dark-light box. Actually, STEP KO mice showed diminished latency to enter the open arms of the plus maze compared to the control group (Table 1), thus indicating that they do not present altered anxiety levels. We also analyzed active vs. passive stress coping using the tail suspension test. STEP KO mice presented reduced latency to immobility (*t*_(1,17)_ = 3.26, Student’s *t*-test; Figure 3A), and increased time of immobility (*t*_(1,17)_ = 2.21, Student’s *t-test*; Figure 3B), pointing at diminished levels of active stress coping.

**Table 1:** Behavioral battery results when evaluating wild-type (WT) and STriatal-Enriched protein tyrosine Phosphatase knockout (STEP KO) mice (*n* = 11–12) in sensorimotor, exploratory and anxiety tests.

	WT	STEP KO	*t*_(1,21)_	*p*<
**Corner test**				
Number of corners	9.42 ± 0.89	6.00 ± 0.77	2.87	0.01
Number of rearings	1.25 ± 0.37	2.45 ± 0.45	2.07	n.s
**Iron rod**				
Muscular strength (latency in s)	9.69 ± 1.59	10.46 ± 2.12	0.29	n.s.
Motor coordination (segments)	1.46 ± 0.61	0.54 ± 0.21	1.37	n.s.
**Wire hanger test—60s**				
Muscular strength (latency in s)	12.05 ± 2.63	6.66 ± 1.40	1.76	n.s.
Motor coordination (segments)	2.00 ± 0.45	1.23 ± 0.45	1.21	n.s.
**Open field**				
Distance (cm)	2185 ± 139.8	1374 ± 217.6	3.19	0.005
Number of groomings	1.42 ± 0.34	2.54 ± 0.39	2.20	0.05
Number of rearings	21.92 ± 2.16	17.27 ± 2.57	1.39	n.s.
Number of defecation boluses	0.58 ± 0.35	2.09 ± 0.47	2.56	0.05
**Plus maze**				
Latency of entry in the open arms (s)	109.7 ± 36.75	21.64 ± 13.93	2.16	0.05
Number of entries in the open arms	3.17 ± 0.82	3.00 ± 0.36	0.18	n.s.
Distance in the open arms (cm)	112.6 ± 29.51	129.1 ± 16.66	0.47	n.s.
% time in the open arms (s)	7.46 ± 1.80	10.79 ± 2.08	−1.21	n.s.
% time in the enclosed arms (s)	78.10 ± 3.39	76.88 ± 2.65	0.28	n.s.
Time in the open arms (s)	22.39 ± 5.42	32.38 ± 6.26	−1.21	n.s.
Time in the enclosed arms (s)	234.30 ± 10.17	230.63 ± 7.97	0.28	n.s.
Number of entries in the open arms	3.16 ± 0.82	3.00 ± 0.35	0.18	n.s.
Number of entries in the enclosed arms	8.41 ± 1.24	7.91 ± 0.49	0.38	n.s.
**Dark-light box**				
Latency of entry into the light compartment (s)	40.69 ± 15.42	60.67 ± 21.85	1.51	n.s.
Number of entrances into the light compartment	5.25 ± 0.69	4.36 ± 0.54	0.99	n.s.
Distance in the light compartment (cm)	258.1 ± 31.53	230.5 ± 32.07	0.61	n.s.
Time in the light compartment (s)	82.39 ± 13.75	46.39 ± 7.86	2.18	0.05

*Results are presented as mean ± SEM. Data was analyzed by Student’s t-test. n.s., non significant*.

### STEP KO Mice Show Altered Social Patterns

**Figure 4:**
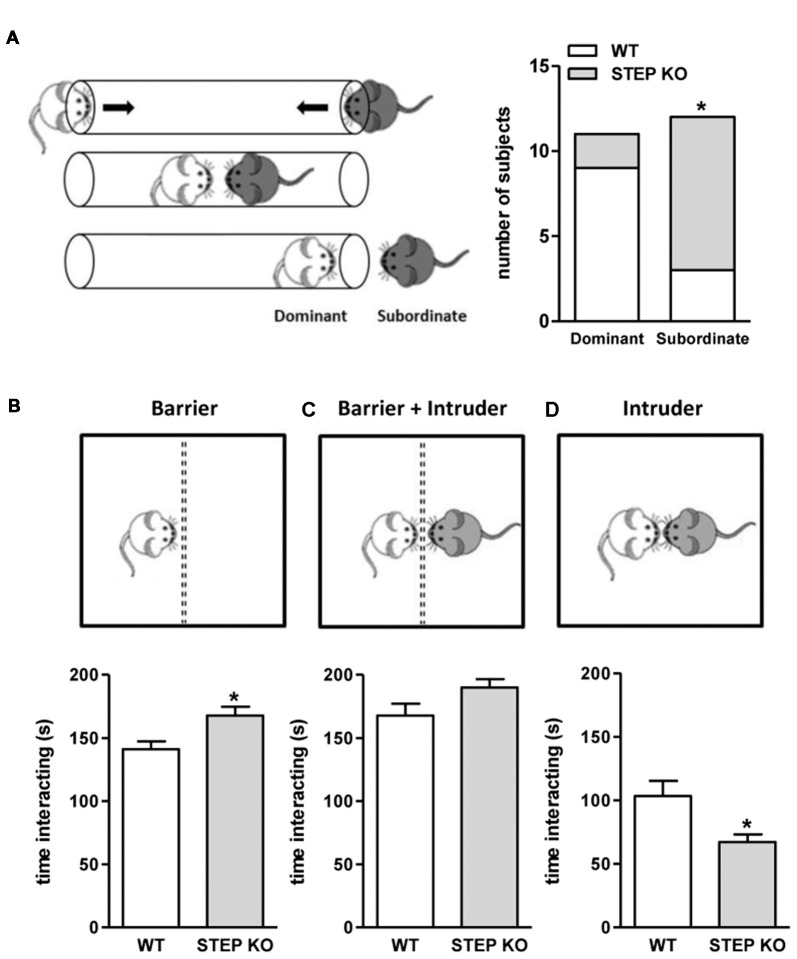
STEP KO mice display altered social patterns. **(A)** Dominant and subordinate behavior of WT and STEP KO (*n* = 11–12) mice in the dominance tube test. Results are presented as number of subjects presenting each phenotype. *p < 0.05 “genotype” effect (Fisher’s test). Time interacting with the barrier **(B)**, with the intruder behind the barrier **(C)** and directly with the intruder **(D)** during the resident-intruder test in WT and STEP KO mice. Results are presented as mean ± SEM (*n* = 11–12). *p < 0.05 “genotype” effect (Student’s independent *t*-test).

Since mice with a genetic deletion of STEP displayed social memory impairment that could not be explained by the presence of olfactory defects, alterations in sensorimotor functions or increased anxiety levels, we next sought to analyze whether social patterns were also affected. For that, we used the dominance tube test and the resident-intruder test. Data from the dominance tube test showed that the number of animals with subordinate behavior was higher in STEP KO mice group compared to controls (Figure 4A). The results from the resident-intruder test indicated that STEP KO mice showed more interest in interacting with the barrier than WT mice during the habituation to the barrier (“genotype” effect *t*_(1,21)_ = 7.31, *p* < 0.05; Figure 4B), but not when the intruder was introduced in the home cage behind the barrier (“genotype” effect *t*_(1,21)_ = 2.86, n.s.; Figure 4C). Nevertheless, and in line with the subordinate behavior observed in the dominance tube test, when the barrier was removed STEP KO mice showed reduced time of interaction with the intruder compared to the WT group (“genotype” effect *t*_(1,21)_ = 6.44, *p* < 0.05; Figure 4D).

### STEP KO Mice Show Increased Basal DA Release in the Dorsal Striatum

**Figure 5:**
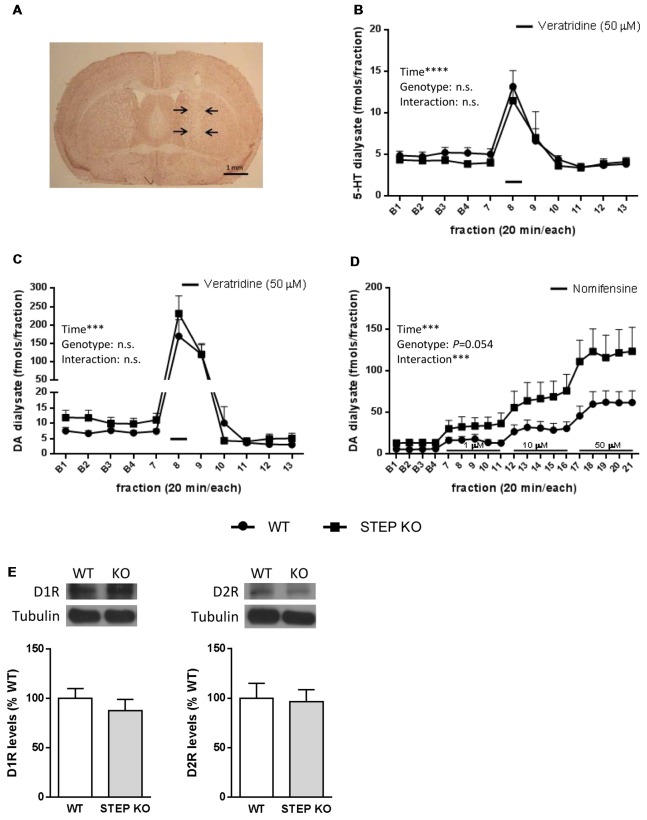
Basal dopamine (DA) release in the dorsal striatum is increased in STEP KO mice. **(A)** Representative photomicrograph illustrating the location (arrows) of the dialysis probe after staining with neutral red. **(B)** Effect of local perfusion of 50 μM veratridine (line) on 5-HT output in the dorsal striatum of WT and STEP KO mice (two-way ANOVA, significant effect of time; ****p < 0.001). **(C)** Effect of local perfusion of 50 μM veratridine (line) on DA output in the dorsal striatum of WT and STEP KO mice (two-way ANOVA, significant effect of time; ***p < 0.001). **(D)** Effect of local perfusion of 1, 10 and 50 μM nomifensine (line) on DA output in the dorsal striatum of WT and STEP KO mice (two-way ANOVA, significant effect of time and time × genotype interaction; ***p < 0.001). Values are expressed as mean 5-HT/DA concentration (fmols/fraction) ± SEM (*n* = 9–11 mice). **(E)** DA D1 receptor (D1R) and DA D2 receptor (D2R) levels were analyzed by Western blot of protein extracts obtained from the striatum of WT and STEP KO mice. Representative immunoblots are shown. Values (obtained by densitometric analysis of Western blot data) are expressed as percentage of WT mice and shown as mean ± SEM (*n* = 10–11). Data were analyzed by Student’s independent *t*-test. n.s., non significant.

Finally, we wondered whether the changes found in social behaviors were accompanied by alterations in neurotransmitter levels in the striatum of STEP KO mice. To answer this question, we implanted a dialysis probe in the dorsal striatum of freely moving WT and STEP KO mice (Figure 5A) to perform *in vivo* microdialysis, and determine extracellular 5-HT and DA levels by HPLC. Basal 5-HT levels (fmols/fraction) were 5.34 ± 0.51 in WT (*n* = 11) and 4.67 ± 0.55 in STEP KO mice (*n* = 11; *t*_(1,20)_ = 0.8933; *p* = 0.3823), and basal 5-HT metabolite 5-hydroxyindoleacetic acid (5-HIAA) levels (pmols/fraction) were 1.27 ± 0.18 in WT (*n* = 11) and 1.27 ± 0.16 in STEP KO mice (*n* = 11; *t*_(1,20)_ = 0; *p* = 1.00). The analysis of the effect of local perfusion of the depolarizing drug veratridine (50 μM) on 5-HT levels indicated a significant effect of time (*F*_(13,247)_ = 13.96; *p* < 0.0001), but no genotype effect (*F*_(1,19)_ = 0.6227; *p* = 0.4398) or time × genotype interaction (*F*_(13,247)_ = 0.3106; *p* = 0.9901; Figure 5B). Thus, both basal levels and veratridine-induced release of 5-HT were similar in the dorsal striatum of WT and STEP KO mice.

On the other hand, basal extracellular DA levels (fmols/fraction) were 6.77 ± 1.00 in WT (*n* = 15) and 11.81 ± 2.76 in STEP KO mice (*n* = 13; *t*_(1,26)_ = 2.326; *p* < 0.05), and basal DA metabolite 3,4-dihydroxyphenylacetic acid (DOPAC) values (pmols/fraction) were 1.22 ± 0.16 in WT (*n* = 15) and 2.15 ± 0.25 in STEP KO mice (*n* = 13; *t*_(1,26)_ = 3.223; *p* < 0.01). Thus, STEP KO mice had significantly increased basal extracellular concentrations of DA and DOPAC in the dorsal striatum compared to WT mice. Perfusion of veratridine (50 μM) increased DA output in the dorsal striatum similarly in both genotypes. Thus, two-way ANOVA showed a significant effect of time (*F*_(10,190)_ = 33.44; *p* < 0.001), but no genotype effect (*F*_(1,19)_ = 0.657; n.s.) or time × genotype interaction (*F*_(10,190)_ = 0.670; n.s.; Figure 5C). The local perfusion of the norepinephrine-DA reuptake inhibitor nomifensine (1, 10 and 50 μM) in the dorsal striatum produced an enhanced DA output in STEP KO mice compared to WT mice. Two-way ANOVA showed a significant effect of time (*F*_(18,306)_ = 24.97; *p* < 0.001), a quasi-significant effect of genotype (*F*_(1,17)_ = 4.254; *p* = 0.054) and a significant time × genotype interaction (*F*_(18,306)_ = 2.929; *p* < 0.001, Figure 5D), pointing at an increased release of DA in the STEP KO mice compared to the WT group. However, STEP KO mice did not show alterations in D1R or D2R striatal levels respect to WT mice (D1R: *t*_(1,19)_= 0.83, n.s.; D2R: *t*_(1,18)_ = 0.18, n.s., Figure 5E).

## Discussion

It has been reported that STEP KO mice show improved cognitive performance in the Morris water maze and in the radial arm maze (Venkitaramani et al., 2011) as well as increased fear conditioning in the conditioning suppression food-motivated instrumental performance test (Olausson et al., 2012). The increased tyrosine phosphorylation of STEP substrates, and downstream targets, in distinct brain regions likely provides a potential molecular mechanism for those results (Venkitaramani et al., 2009, 2011; Zhang et al., 2010; Olausson et al., 2012). Indeed, based on previous works (Suzuki et al., 2011; Sinai et al., 2012), it could be expected that STEP KO mice show improved social memory. However, social memory and social behaviors have been barely studied in this mouse model, and there are also reports showing that alterations in the ERK pathway may underlie altered social behavior (Satoh et al., 2011; Faridar et al., 2014). In the present study, we focused on the social phenotype of STEP KO mice, and we show that STEP deficient mice present impaired social memory. Since WT and STEP KO mice showed similar levels of sociability (present results; Venkitaramani et al., 2011; Goebel-Goody et al., 2012b), and non-social hippocampal-dependent learning and memory is intact or improved (present results; Venkitaramani et al., 2011; Sukoff Rizzo et al., 2014; Castonguay et al., 2018), our results indicate that STEP deficiency specifically disturbs social memory.

In contrast to the present findings, a previous work reported that STEP KO mice had intact social memory (Venkitaramani et al., 2011). In that study, only a direct interaction test was performed, a smaller group of animals was tested, and data of social memory assessment was not provided (Venkitaramani et al., 2011). However, and in accordance with our results, these authors did report data of time spent in social interaction pointing at intact sociability levels in STEP KO mice. Strikingly, in another study, neither WT nor STEP KO mice showed social memory in the three-chamber sociability test (Goebel-Goody et al., 2012b). Probably, the lack of social memory in WT mice was due to the stimulus mice being from mixed genotypes. Indeed, it has been reported that social communication is affected by genotype, since social signaling from mice other than WT may elicit unusual social behaviors in the subject mice (Wood and Morton, 2015). To eliminate this confounding variable, in the present study all the stimulus mice used to assess social memory were WT animals.

As far as we have investigated, an impaired olfactory system or altered anxiety levels in STEP KO mice are unlikely to underlie the social memory deficits reported here. Indeed, STEP KO mice behave like WT mice in the olfactory habituation/dishabituation test, and no significant differences were observed in the plus maze or the dark-light box tests compared to WT mice, as previously described (Goebel-Goody et al., 2012b). In addition, STEP KO mice spent significantly more time in the center of the open field than WT mice, and did not display anxiety-related phenotypes in the stress induced hyperthermia test (Sukoff Rizzo et al., 2014).

The present results indicate that STEP KO mice consistently show reduced exploratory activity in the open field, in the socialization and test phases of the three-chamber sociability test, and in the habituation phase of NORT. Although this finding could have a confounding effect on the outcome in social tasks, several results suggest that this was not the case. During the socialization phase in the three-chamber sociability test, STEP KO mice explored the mouse similarly to WT mice, indicating similar levels of interest for social novelty as well as similar time for social learning acquisition compared to WT mice. In addition, the results obtained in the first trial of the social discrimination test and the 11-trial habituation/dishabituation test and their learning curves point to a similar interest and similar time for acquisition of a new social stimulus in both genotypes. Altogether, these results strengthen the idea that altered locomotion in STEP KO mice is unlikely to influence their outcome in social memory tests. In contrast to the present findings, previous data documented that STEP KO mice do not present alterations in spontaneous exploratory activity compared to controls (Venkitaramani et al., 2011; Sukoff Rizzo et al., 2014; Legastelois et al., 2015). Given that STEP KO mice have intact motor capabilities (present results) and motor coordination (Venkitaramani et al., 2011; Sukoff Rizzo et al., 2014), this discrepancy might be explained by differences in methodological procedures. In our study, we assessed exploratory activity in a 5- and 10-min test, respectively, where a fear-to-novelty component of the first minutes might be diminishing STEP KO exploratory activity. In contrast, the previous studies evaluated longer trials, and thus the initial fear-to-novelty effect is likely diluted. In fact, it is noteworthy that the analysis of the initial 5–10 min in the open field reported in earlier studies suggests that STEP KO mice present a trend toward reduced exploratory activity respect to the control group (Venkitaramani et al., 2011; Sukoff Rizzo et al., 2014; Legastelois et al., 2015). Further supporting our hypothesis, the data of the corner test also pointed at increased fear-to-novelty in STEP KO mice. In agreement with our findings, it was reported that STEP deficient mice were hypoactive in the social novelty phase of the three-chamber test, where behavior is also evaluated in trials of 10 min duration (Goebel-Goody et al., 2012b). Actually, in a mouse model of fragile X syndrome, increased STEP levels promote locomotor hyperactivity that can be prevented by genetic deletion (Goebel-Goody et al., 2012b) or pharmacological inhibition of STEP using TC-2153 (Chatterjee et al., 2018). Nevertheless, no differences between STEP KO and WT group were found in a 5 min trial in the open field, probably due to the wide range of ages of the subjects (Goebel-Goody et al., 2012b). Overall, altered locomotion in STEP KO mice does not seem to play a major role in the social phenotype reported here, and STEP KO mice showed intact social memory in the form of habituation as indicated by the learning curves. However, STEP KO mice did show a compromised performance in the test phase. Nonetheless, we cannot rule out the contribution of lack of dishabituation to a new stimulus in the absence of STEP.

Neurotransmitters like 5-HT and DA are involved in the brain circuitry related to social behaviors (Miczek et al., 2002; Watanabe and Yamamoto, 2015; Lu et al., 2018). The dorsal striatum plays a role in internally guided social behavior, the ventral striatum regulates social behavior by the integration of external social stimuli (Báez-Mendoza and Schultz, 2013; van den Bos, 2015), and the balance of 5-HT and DA levels determines the role of each striatal region in social behaviors (van den Bos, 2015). Here, we reported that extracellular levels of 5-HT in the dorsal striatum are similar in both genotypes. However, we observed significantly increased basal extracellular concentrations of DA, and higher DA output in response to inhibition of DA reuptake in the dorsal striatum of STEP KO mice, without alterations in the total levels of D1R and D2R. The molecular mechanism underlying this result is currently unknown, but it could be related to the finding that phosphorylated Pyk2^Tyr402^, a STEP substrate (Xu et al., 2012), has been implicated in DA release in PC12 cells (Zhang et al., 2014, 2016). Moreover, phosphorylation of synapsin I, which regulates the probability of vesicle release (Cesca et al., 2010), was found to be increased in STEP KO mice (Venkitaramani et al., 2011; Bosco et al., 2018).

It has been shown that high levels of DA in the dorsal striatum increase motivation, approach and reward behavior (Ikemoto et al., 2015), and social motivation and reward behaviors include those that happen in a social context (Báez-Mendoza and Schultz, 2013). Surprisingly, our data suggest that STEP KO present decreased interest/motivation as shown by the reduced exploratory activity (as discussed above), diminished active coping to stress in the tail suspension test, reduced exploration of the intruder in the resident intruder test, as well as diminished exploration of a new home cage in the corner test. Thus, our findings suggest that STEP KO mice present decreased motivation to cope with new environmental or social stimuli. On the other hand, it has been reported that mouse models with altered dopaminergic neural state also present social dominance and aggressive behavior (Rodriguiz et al., 2004; Adamczyk et al., 2012; McNamara et al., 2017). However, despite the presence of higher DA levels in STEP KO mice, they presented reduced dominance behavior in the dominance tube test and interacted less time with the intruder mouse in the resident-intruder test. Conversely, a previous study found that STEP KO and WT mice retreat from the social dominance tube test at a similar frequency (Goebel-Goody et al., 2012b), and the initial characterization of STEP KO mice reported greater dominance behavior than control mice scored based on holding down the other mouse against the cage floor or cage wall (Venkitaramani et al., 2011). It should be kept in mind that global genetic manipulation of STEP levels may promote compensatory mechanisms or developmental modifications. In fact, an acute pharmacological inhibition of STEP after intraperitoneal injection of 10 mg/Kg TC-2153 (Xu et al., 2014) in WT mice had no effect in most parameters analyzed in the open field, light-dark box task or three-chamber social test (Chatterjee et al., 2018). Moreover, we cannot rule out the contribution of other brain regions and neurotransmitters (van Erp and Miczek, 2000; Miczek et al., 2002). For instance, glutamate release is also increased in STEP deficient mice (Bosco et al., 2018), and it is known that glutamate and DA neurotransmission modulate each other in the striatum (Mora et al., 2008; Gardoni and Bellone, 2015).

In conclusion, the present results highlight that lack of STEP activity impairs social memory in the absence of affected olfactory function or altered anxiety levels, and produces changes in social patterns accompanied by dysregulation of striatal DA homeostasis.

## Author Contributions

GB, AS and EP-N conceptualized the study, and EP-N supervised it. GB designed the behavioral phenotyping, performed the experiments, data analysis and interpretation of results. AC designed the microdialysis studies, performed the experiments with GB and MM, and analyzed, interpreted data of microdialysis experiments, and wrote this section. GB and AS interpreted the results, wrote the manuscript and prepared the figures. All the authors critically reviewed the content and approved the final version.

## Conflict of Interest Statement

The authors declare that the research was conducted in the absence of any commercial or financial relationships that could be construed as a potential conflict of interest.
