# Supplementary material for: Social Memory and Social Patterns Alterations in the Absence of STriatal-Enriched Protein Tyrosine Phosphatase
Source: Front Behav Neurosci. 2019 Jan 25;12:317. doi: 10.3389/fnbeh.2018.00317 (PMC6362413; doi:10.3389/fnbeh.2018.00317)
Supplement: Supplementary file 1 [file Data_Sheet_1.PDF]

Social memory and social patterns alterations in the absence of STriatal-Enriched protein tyrosine Phosphatase

Gloria Blázquez[1-3], Anna Castañé[2,4,5], Ana Saavedra[1-3], Mercè Masana[1-3], Jordi Alberch[1-3], Esther Pérez-Navarro[1-3]*

[1]Departament de Biomedicina, Facultat de Medicina i Ciències de la Salut, Institut de Neurociències, Universitat de Barcelona, Barcelona, Catalonia, Spain
[2]Institut d'Investigacions Biomèdiques August Pi i Sunyer (IDIBAPS), Barcelona, Catalonia, Spain
[3]Centro de Investigación Biomédica en Red sobre Enfermedades Neurodegenerativas (CIBERNED), Spain
[4]Department of Neurochemistry and Neuropharmacology, CSIC-Institut d'Investigacions Biomèdiques de Barcelona (IIBB), Barcelona, Catalonia, Spain
[5]Centro de Investigación Biomédica en Red de Salud Mental (CIBERSAM), Spain

To whom correspondence should be addressed: estherperez@ub.edu

Figure S1

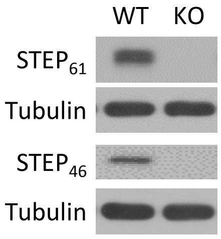

FIGURE S1. Mouse genotype was confirmed after behavioral analyses. STEP levels were analyzed by Western blot of protein extracts obtained from the striatum of WT and STEP KO mice. Representative immunoblots showing the presence of $STEP_{61}$ and $STEP_{46}$ in WT, but not in STEP KO mice.

Figure S2

Batch 1

SHIRPA OF PM NORT DLB 3-CST DT RIT →

Batch 2

11-HDT SDT OHDT TST →

FIGURE S2. Timeline of the behavioral analyses performed in WT and STEP KO mice. Two batches of animals were used (n=10 WT mice and n=9-11 STEP KO mice each batch). OF, open field; PM, plus maze; NORT, novel object recognition test; DLB, dark-light box; 3-CST, three-chamber sociability test; DT, dominance tube; RIT, resident-intruder test; 11-HDT, 11-trial habituation/dishabituation social recognition test; SDT, social discrimination test; OHDT, olfactory habituation/dishabituation test; TST, tail suspension test.

Figure S3

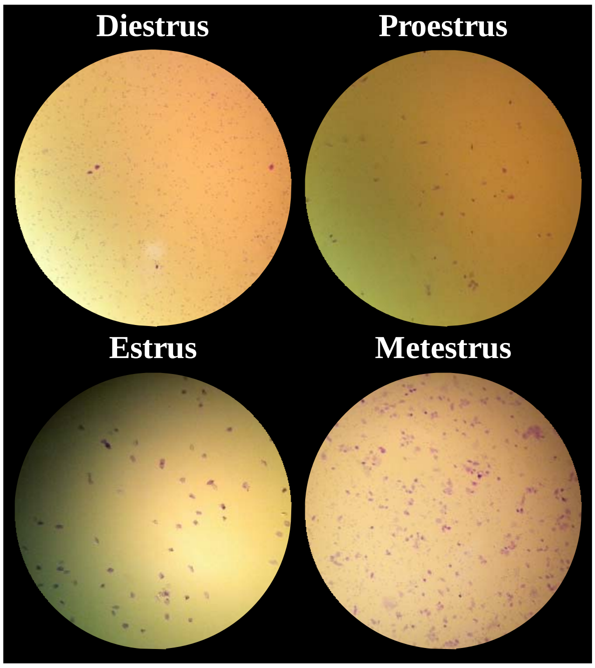

FIGURE S3. Representative photomicrographs of vaginal smears after staining with crystal violet to illustrate the four phases of oestrus cycle. Females in diestrus and metestrus were used as stimulus females in the 11-trial habituation/dishabituation test.

Figure S4

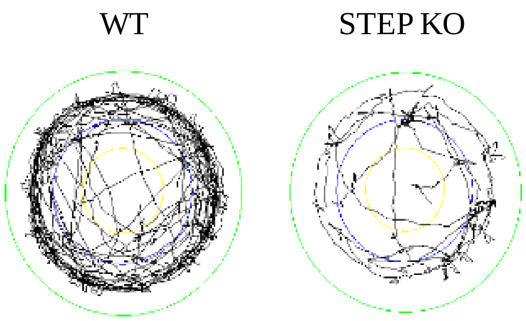

FIGURE S4. Representative images of open field path length from WT and STEP KO mice during a 5 min trial.
